# Vibratory behaviour produces different vibration patterns in presence of reproductives in a subterranean termite species

**DOI:** 10.1038/s41598-021-88292-7

**Published:** 2021-05-10

**Authors:** Louis Pailler, Samuel Desvignes, Fanny Ruhland, Miguel Pineirua, Christophe Lucas

**Affiliations:** Institut de Recherche Sur La Biologie de L’Insecte (UMR7261), CNRS – University of Tours, Tours, France

**Keywords:** Ecology, Evolution, Zoology

## Abstract

Vibratory behaviours are widespread in social insects, but the produced vibrations remain poorly explored. Communication using vibrations is an efficient way to transmit information in subterranean environments where visual and odorant signals are less efficient. In termites, different vibratory behaviours are performed in different contexts like reproductive regulation and alarm signalling, but only few studies explored the structure of the produced vibrations (i.e., duration, number of pulses, amplitude). Here, we described several types of vibrations produced by a vibratory behaviour widespread in termites (body-shaking), which can be transmitted through the substrate and detected by other colony members. We analysed the structures of the emitted vibrations and the occurrence of the body-shaking events in presence/absence of reproductives and/or in presence/absence of a stress stimuli (flashlight) in the subterranean termite *Reticulitermes flavipes*. Interestingly, only the presence of the reproductives did influence the number of pulses and the duration of the emitted vibrations. Moreover, the first part of the emitted vibrations seems to be enough to encode reproductive information, but other parts might hold other type of information. Body-shaking occurrence did increase in presence of reproductives but only briefly under a flashlight. These results show that vibratory cues are complex in termites and their diversity might encode a plurality of social cues.

## Introduction

Communication is a central component of all animal behaviours allowing the transfer of vital information during interactions. There are different ways to transmit information including tactile, visual, chemical, and vibroacoustic cues between an emitter and a receiver^1–3^. The usage of these communication modes depends on the context and are complementary. It is even more complex for social animals because sociality involves frequent tight communications between and among individuals of the group. In eusocial insects, signals are diversified and fit to the plurality of the social interactions involved in the colony functioning, i.e. nestmates recognition, reproductive status signalling, nest defence, alarm behaviour, recruitment, foraging behaviour^4^. Signals could also be multimodal, i.e., a single signal could be transmitted using different forms of communication, and may activate different sensory systems^5–7^. Additional channels used to transmit multimodal signals afford others possibilities of communication^8^. For example, alarm communication in termites involves a mix of chemical and vibratory signals, where the alarm pheromones released by soldiers increased vibratory signals from workers^9^.

In eusocial insects, signals modulation allows to increase the diversity of the coded information inside each communication canal. For example, chemical communication uses different compounds which are produced by the colony members according to their role and the current social context. The hydrocarbon profiles of bees, wasps, ants, and termites vary in function of the individual fertility and their dominance status^10^. The number of example in chemical communication is vast^11,12^ but other communication canals are also involved and modulated like visual cues^13^. Another communication canal present in ants, bees, wasps and termites, is used to transfer vibratory-acoustic cues^14–18^. These cues are widespread in termites with several behaviours able to produce vibrations like head-butting^19–22^, chewing^17^ and body-shaking^9,23^. For example, the species *Cryptotermes secundus*, use the resonance of their own vibrations to assess wood quality and recruit foragers^17^. Modulation of vibratory behaviours is also used as an alarm signal^21,24^ or during cannibalism^23,25,26^. Many termites are lucifugous^27^ and in several species, individuals increase their vibratory behaviours like body-shaking or head-drumming (the head hits the ground) when exposed to a flashlight^9,20^, a stimulus considered as aggressive for these species^24,28^. The social context, as the caste ratio or the presence of reproductives can also induces modulation of vibratory behaviours^29^.

The social context is one of the most relevant factors influencing the complex communication system involved in the colony functioning of social insects. Among them, the presence or absence of reproductives affects the physiology and behaviour of the other colony members, and therefore represents one of the essential components influencing the social stability of the colony. Access to the reproductive status is regulated through social interactions with specific chemical signals^30,31^ and with expression of specific genes, also affecting non-reproductive individuals^32,33^. The reproductive status could also be encoded by vibrations. In the social wasps *Polistes biglumis*, abdominal wagging behaviours (a vibratory signal) are produced by foundresses only in the period before emergence of offspring. Furthermore, the number of vibratory signals is dependent on the colony’s cast ratio, and could influence larval development and production of workers^29^. In termites, vibrational communication could also be dependent of the presence/absence of reproductives or their proxy (eggs or royal pheromone)^34–36^.

Among vibratory behaviours performed by termites, the contexts triggering the body-shaking (also called longitudinal vibrations, LOM, jerking, jittery movements, jigging, shaking, tremulation or trembling^22–25,37–40^). appeared to be diverse like alarm signals^9,24^, cannibalisms^41^, and more recently it appeared to be correlated with reproductives signalling^36,42^. The body-shaking behaviour corresponds to a back-and-forth longitudinal oscillatory movement of the entire body^20^. However, the vibrations produced during a body-shaking are barely known. A first attempt to characterize the emitted vibrations was done recently by Delattre et al*.*^9^. They measured the speed of motion in a mix group of workers and soldiers and showed that the global activity of the box generated vibrations. Nevertheless, no information was available to directly connect an individual performing a body-shaking with the emitted vibrations produced by this behaviour event. To go further, only a clear description of the structure of the emitted vibrations (i.e. duration, number of pulses, amplitude) with a direct association with body-shaking events, would allow to unravel the potential diversity of the vibrations. This would open the door to functional studies allowing to disentangle its role in alarm signals, cannibalisms, and reproductive signalling, or its potential implication in other unknown social interactions. To do so, we hypothesized that the environmental contexts could influence the structure of the emitted vibrations produced by a body-shaking event. First, we confirmed that emitted vibrations could be measured and transmitted through the substrate, allowing to transport potential information between individuals. Then, we investigated the influence of two different contexts known to induce this behaviour in termites: (1) presence/absence of reproductives (a social context stimulation) and (2) presence/absence of a flashlight (an alarm stimulation). The experiments were done on workers of the subterranean species *Reticulitermes flavipes* (Kollar, 1837), a worldwide invasive termite^43^ known to present a behavioural plasticity of the body-shaking^36^. For each treatment, we analysed in detail the structure of the vibrations emitted through the substrate using a laser vibrometer. At the same time, video recordings allowed to directly associate the emitted vibrations to the expressed behaviours. Our predictions were that vibrations could present specificities in duration of the signal or in the number of pulses, according to the tested contexts (reproductives/flashlight). Finally, we did hypothesize that more body-shaking events will be expressed in presence of reproductives or flashlights than in their absence.

## Material and methods

### Study species and laboratory conditions

Thirteen colonies of the termite *R. flavipes* were collected in Oléron (France) between 2015 and 2018. Colonies were at least 300 m apart distance to ensure of the independence of colonies^43^. Upon collection, colonies were placed in plastic boxes (24 × 18 × 10; multiroir Cat#45105boila) with a fraction of their own nest material, moistened sand, a cellulosic ultrapure paper (47 mm diameters; Whatman, grade 42 Ashless) and supplied with pin wood sawdust^44^. Colonies were maintained under dark conditions (26 ± 1 °C and > 90% RH) within black plastic boxes^45^. To distinguish sexes of reproductives, the site of the seventh posterior sternite was examined under microscope, which is longer in females than in males^46^.

### Experimental setup

**Figure 1:**
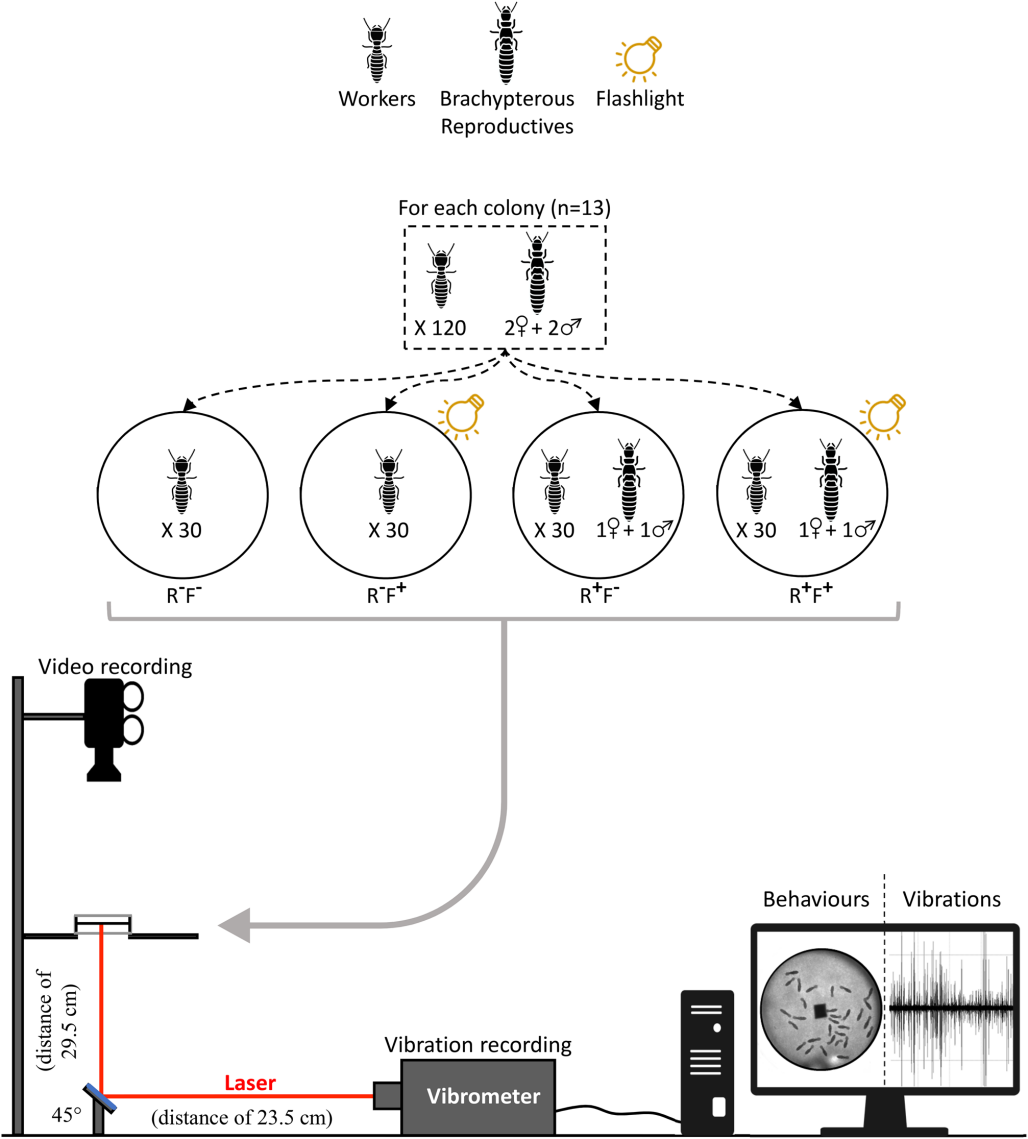
Experimental design representing the different treatments, the video recordings of the behaviour events, and the laser recordings of the emitted vibrations. Workers in presence of reproductives and flashlight **(**R^**+**^F^**+**^), in presence of reproductives **(**R^**+**^F^**−**^), in presence of flashlight **(**R^**-**^F^**+**^), and in absence of reproductives and flashlight **(**R^**-**^F^**-**^).

Body-shaking events and their produced vibrations were investigated in 13 colonies in presence/absence of reproductives (one male with one female of brachypterous neotenics) and with/without a flashlight stimulation (Fig. 1). Thirty workers were selected for each treatment, for a total of 120 workers per colony for the 4 treatments. Note that more individuals per box would have involved more vibrations events produced at the same time. Because we wanted to directly link each body-shaking event with each vibration event, we limited the number of workers to 30 per treatment. Reproductives and workers came from to the same colony (n = 13). One colony (i.e., all four treatments) was realized per day for 13 consecutive days, by first isolating the workers in 4 different plastic boxes (50 mm diameter; Starpack Cat#04913) with moistened pure cellulose paper (47 mm diameter; Whatman, GE Healthcare).

Experimental boxes were then prepared to allow both behavioural recordings and vibration recordings. To maximise propagation of vibrations on the substrate, a pure cellulose paper (47 mm diameter; Whatman, GE Healthcare) were wedged between two plastic rings (4.8 cm × 6 mm × 3.5 mm), forming a tympan-like paper membrane with clamped conditions at the edge. A piece of adhesive reflective paper (4 mm^2^) was pasted in the centre of the cellulose paper. The suspended paper was moistened using 100 μl of pure water (determined as the best humidity ratio for vibration transmission in our pre-tests). Then, workers and reproductives were transferred in experimental boxes (50 mm diameter; Starpack Cat#04913). Each box was transferred to an anechoic chamber in the dark where all the experiments were realized to minimised external noise pollution. Individuals could settle down for 30 min prior to the video recordings. The flashlights were realised with white LED lights (1000 lx) for 3 s. Measures (behaviour and vibration) were recorded at the same time over 5 min with a t zero setup right after the flashlights.

### Behavioural and vibration analyses

Behavioural recordings were performed using an infrared camera (Basler acA1300—60gc) driven by the software Pylon Viewer (v5.0.12.11830) during 5 min. The camera was fixed to a rail above the recorded experimental boxes containing the termites. Two infrared LED lights (Sygonix Mit 48 IR) allowed to record behaviours under dark conditions. Experimental boxes containing the termites were placed at 29.5 cm above a mirror inclined at 45°. The laser beam of the vibrometer was reflected on the mirror to reach the reflective paper placed on the underside of the suspended paper of the experimental boxes. The vibrometer was placed at 23.5 cm from the mirror. Vibration recordings were performed using a vibrometer Polytec PDV 100 driven by the software Vibsoft (v5.2). Vibration recordings were performed during the same 5 min than for the video recordings in real-time, allowing to cross-check vibration events with body-shaking events.

Videos analyses were performed using the freeware BORIS (v7.4.7)^47^ to quantify the number of body-shakings during a period of 5 min. Analyses of the emitted vibrations were performed using the GNU software Octave (v5.1.0). The different structures of the vibrations produced by each body-shaking event were analysed in detail along with the behavioural video recordings. Therefore, we were able to calculate the duration, number of pulses, the frequency, and the average amplitude of the emitted vibrations (which we call thereafter structure of the vibrations). Analyses of the vibration events revealed differences in the frequencies of the emitted vibrations. Some body-shaking were producing vibration events composed of only one series of pulses (thereafter called one part) with one frequency, and other body-shaking were producing vibration events composed of two series of pulses (thereafter called two parts) with two distinctive frequencies. These two types of body-shaking event were called BS1 and BS2, respectively. An example of body-shaking type BS2 is represented in the video of the figure S1, along with the corresponding vibration event composed of two parts. It can be clearly seen in this high-speed recording video at 1000 fps, that BS2 is composed of two different behavioural sequences. Other types of body-shaking composed of three and four parts were also found and called respectively BS3 and BS4. They both present a first part with a different frequency than the other remaining parts with a long-time interval between parts (Fig. S2). Note that a few BS5 and BS6 were also found but were too rare to be analysed (see the result section for details). It allowed to classify the body-shaking events into different types in function of the number of parts they contained. To identify vibration events in the laser recordings using the software Octave (v5.1.0), the amplitude of the background noise was determined to calculate a threshold value used by the software. This threshold value was determined using ten random points for each recording. Peaks above this threshold were considered as a vibration event and were cross checked with the video recordings, to confirm that this vibration event is linked to a unique observed body-shaking event. When more than one body-shaking event were expressed at the same time by different individuals, the vibration event was discarded from future analyses. Data were recorded and analysed blindly regarding the treatments^48^.

### Statistical analyses

The number of body-shaking counted in the video recordings were analysed with the presence/absence of reproductives/flashlights as explanatory factors using a general linear mixed model (LMM). When the time was used as an explanatory variable and were analysed along with the body-shaking events, a generalized linear mixed model (GLMM) with Poisson error was used. For this analysis, we first entered the time (categorial data) and the presence of reproductives as explanatory variables. The shortest time-lapse allowing all the 13 colonies to be still represented in the dataset was determined to be a 15-s time-lapse. The reproductive effect was always significant for all the tested time-lapse. Because of the significant effect of the presence of reproductives and the non-significance of the interactions between the time and the presence of reproductives, the dataset was then split per presence/absence of reproductives. The two resulting subsets were used to conduct two additional GLMM with time and presence/absence of flashlight as explanatory factors. Analyses of the number of body-shaking classified into the different types were done using GLMM with Poisson error distribution. The dataset was split into two subsets, with and without flashlight, using the body-shaking types and the presence/absence of reproductives as explanatory factors. The frequency of the emitted vibrations was analysed using LMM with the type of body-shaking, the flashlight, and the presence of reproductives as explanatory factors. Due to the significant effect of the type of body-shaking and the presence of reproductives on the frequency, we investigated separately the duration and the number of pulses. The structures of the emitted vibrations (total duration, number of pulses and amplitude) were analysed using LMMs with the type of body-shaking, the flashlight, and the presence of reproductives as explanatory factors. When the interaction between the three factors was significant, the different types of body-shaking were analysed separately. All the different parts of each type of body-shaking were analysed and compared separately or in interactions, but for clarity purpose only significant ones were represented in the figures (Fig. 6). The flashlight was also removed from the analyses when it had no significant effect. Please note that some non-significant interactions are reported anyway to allow direct comparison between figures. Nevertheless, all tested effects and *p* values are reported in Table 1.

**Table 1:** Statistical table of the tested effects: the type of body-shaking (BS1-4), the presence of reproductives, the flashlight, and all their interactions.

	All parts		First parts		Second parts		Third parts		Fourth parts		Last parts only	
	LR χ^2^	*p*	LR χ^2^	*p*	LR χ^2^	*p*	LR χ^2^	*p*	LR χ^2^	*p*	LR χ^2^	*p*
**(a) Duration**												
Types	**82.96**	**< 0.0001**	**60.16**	**< 0.0001**	**132.09**	**< 0.0001**	**92.70**	**< 0.0001**	x	x	**78.35**	**< 0.0001**
Reproductives	0.19	0.662	**3.94**	**0.047**	0.06	0.801	0.72	0.396	2.79	0.094	1.23	0.267
Flashlight	0.90	0.342	0.90	0.330	0.001	0.972	0.44	0.506	0.07	0.797	0.70	0.403
Types:Reproductives	–	–	–	–	1.13	0.567	–	–	–	–	–	–
Types:Flashlight	–	–	–	–	0.55	0.758	–	–	–	–	–	–
Reproductives:Flashlight	–	–	–	–	0.85	0.358	–	–	–	–	–	–
Type:Reproductive:Flashlight	–	–	–	–	**6.05**	**0.049**	–	–	–	–	–	–
**(b) Number of pulses**												
Types	**307.73**	**< 0.0001**	**28.65**	**< 0.0001**	3.75	0.153	2.86	0.091	x	x	**204.54**	**< 0.0001**
Reproductives	**4.28**	**0.039**	**7.85**	**0.005**	0.62	0.432	1.59	0.208	0.38	0.536	2.60	0.107
Flashlight	0.77	0.380	1.36	0.244	0.45	0.504	0.0017	0.967	0.002	0.962	0.001	0.975
Types:Reproductives	–	–	–	–	–	–	–	–	–	–	–	–
Types:Flashlight	–	–	–	–	–	–	–	–	–	–	–	–
Reproductives:Flashlight	–	–	–	–	–	–	–	–	–	–	–	–
Type:Reproductive:Flashlight	–	–	–	–	–	–	–	–	–	–	–	–
**(c) Amplitude**												
Types	**15.10**	**0.002**	3.22	0.358	0.31	0.858	1.54	0.215	x	x	**20.74**	**< 0.001**
Reproductives	1.91	0.168	1.63	0.201	1.17	0.280	0.35	0.552	0.65	0.419	2.19	0.139
Flashlight	0.85	0.357	1.97	0.160	0.002	0.967	0.13	0.720	1.52	0.218	1.22	0.270
Types:Reproductives	–	–	–	–	–	–	–	–	–	–	–	–
Types:Flashlight	–	–	–	–	–	–	–	–	–	–	–	–
Reproductives:Flashlight	–	–	–	–	–	–	–	–	–	–	–	–
Type:Reproductive:Flashlight	–	–	–	–	–	–	–	–	–	–	–	–

Each effect is reported for (a) the duration, (b) the number of pulses, and (c) the amplitude. Data are shown with all parts pooled together (the entire vibration events), the first parts only, the last parts only, and each part separately. Significant *p* value are in bold.

**Figure 2:**
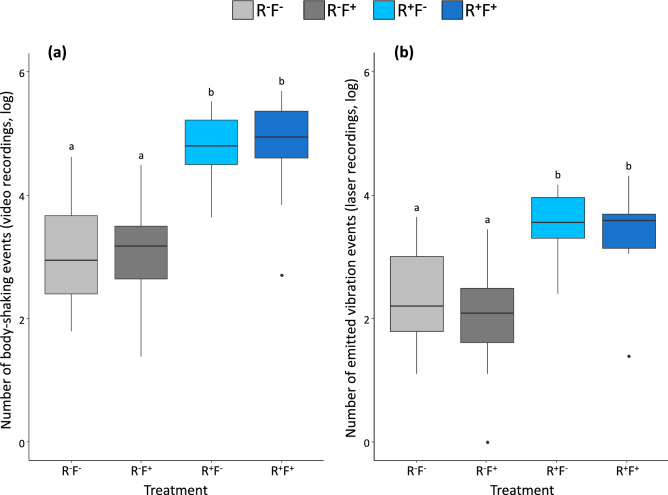
Total number of body-shaking events during video recordings (**a**) and total number of the emitted vibrations measured by laser recordings (**b**) in the four different treatments: absence of reproductives (R^**−**^), presence of reproductives (R^**+**^), absence of flashlight (F^**−**^), presence of flashlight (F^**+**^). Different letters refer to *p* < 0.05.

Colonies were included as random factors in all the above statistical models. The total number of body-shaking events per treatments (shown in Fig. 2), the total duration and the number of pulses of the different types of body-shaking (Fig. 6) were log-transformed to fit with homoscedasticity and normal distribution of model residuals. First, all possible interactions among explanatory variables were tested for all the models before to be simplified step‐by‐step by removing the non‐significant interactions. Post hoc pairwise comparisons were conducted when required using model contrasts and corrected for multiple testing using Tukey corrections. All analyses were performed using the software R v3.6.1 (www.r-project.org) loaded with the packages *lme4*, *car* and *multcomp*.

## Results

Among all the recorded vibrations, 29.1% of them were considered as unique vibration events because of their direct connections with a unique body-shaking event (thanks to the video recordings). To prevent misinterpretation, all none-unique vibration events were excluded from the analyses. Figure 2 shows that both body-shaking occurrences (all the vibratory behaviour observed in video recordings, Fig. 2a) and vibration events (laser recordings which are associated with a unique body-shaking event, Fig. 2b) present a similar trend. They both significantly increase in presence of reproductives (respectively: Fig. 2a, χ^2^ = 102.18, *p* < 0.0001 and Fig. 2b, χ^2^ = 46.37, *p* < 0.0001) independently from flashlight stimulation (respectively: Fig. 2a, χ^2^ = 0.0001, *p* = 0.991 and Fig. 2b, χ^2^ = 2.23, *p* = 0.134). These results permit to ensure that body-shaking events (expressed behaviour) give the same results than laser recordings (emitted vibrations during body-shaking events).

**Figure 3:**
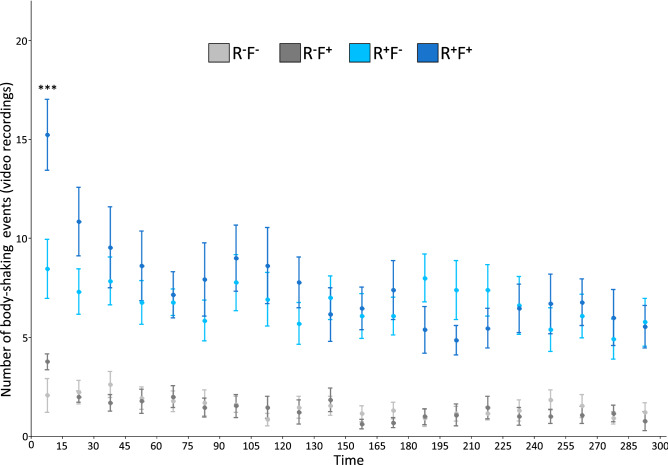
Total number of the body-shaking events (video recordings) over time, with a timeframe set to 15 s. Treatments: absence of reproductives (R^**−**^), presence of reproductives (R^**+**^), absence of flashlight (F^**−**^), presence of flashlight (F^**+**^). Significant effects of the flashlight are represented with * (***p < 0.001). Note that these models were restricted to the comparison within R+ (blue/light blue) or R− (dark grey/light grey) only.

Analyses overtime show that the number of body-shaking events (video recordings) were higher in presence of reproductives (χ^2^ = 1509.51, *p* < 0.0001) through the whole-time interval (Fig. 3). In presence of reproductives (R^+^), the number was dependent on an interaction between the time and the presence of flashlight (χ^2^ = 59.61, *p* < 0.0001) with a higher number, only at 15 s after the flashlight (t = 4.947, *p* < 0.01). For all the other time points, flashlight had no effect (all *p* > 0.5334). In absence of reproductives (R^-^), the number of body-shaking events (video recordings) were dependent on the time (χ^2^ = 84.86, *p* < 0.0001) but not the flashlight (χ^2^ = 0.64, *p* < 0.425).

**Figure 4:**
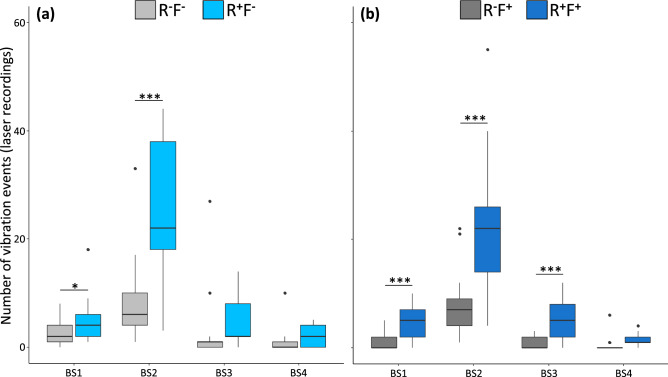
Total number of the vibration events (laser recordings) for the different types of body-shaking (BS1-4). Treatments: absence of reproductives (R^**−**^), presence of reproductives (R^**+**^), absence of flashlight (F^**−**^), presence of flashlight (F^**+**^). Datasets are regrouped in presence (**a**) or absence (**b**) of flashlight. Significant effects of the presence of the reproductives are indicated by stars (*p < 0.05; ***p < 0.001). Dots indicate outliers.

Our data showed that vibrations produced by body-shaking events are measurable and transmitted through the substrate in our experimental setup. A single vibration event is powerful enough to reach the maximum range of the box, which is 2.5 cm (radius), and therefore could be detectable by all the other individuals in the box. Laser analyses of those vibrations allowed to identify 6 different types of body-shakings with specific structure composed of distinctive parts, ranging from one part (BS1) to six parts (BS6). Due to the low number of emitted vibrations recorded with a fifth or sixth part (twelve BS5 and one BS6 respectively over a total of 1306 recording events), analyses were conducted only on body-shaking with four parts or less. Emitted vibrations with two parts were the most abundant. Examples of the four types of body-shaking with their different parts are represented in the supplementary Fig. 2. The number of each type of body-shaking events were different with and without the presence of flashlight (Fig. 4). Indeed, the interaction between the presence of reproductives and the type of body-shaking was significant without flashlight (Fig. 4a: χ^2^ = 11.07, *p* = 0.011) but only marginally significant in presence of flashlight (Fig. 4b: χ^2^ = 7.0545, *p* = 0.070). For both data set (without and with flashlight; Fig. 4a and 4b respectively), the presence of reproductives increase the number of BS1 (Fig. 4a: Z = 3.243, *p* = 0.023; Fig. 4b: Z = 4.786, *p* < 0.001), BS2 (Fig. 4a: Z = 9.912, *p* < 0.001; Fig. 4b: Z = 9.5, *p* < 0.001), and were similar for BS4 (Fig. 4a: Z = 1.702, *p* = 0.658; Fig. 4b: Z = 1.917, *p* = 0.491). Interestingly, the number of BS3 was significant only in presence of flashlight (Fig. 4a: Z = 2.005, *p* = 0.447; Fig. 4b: Z = 5.686, *p* < 0.001).

**Figure 5:**
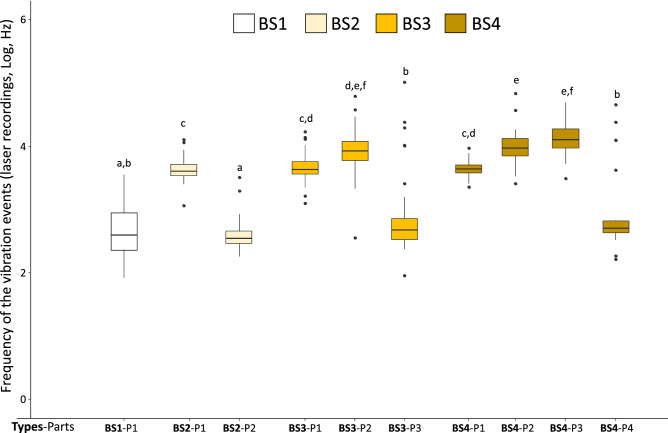
Frequency of the vibration events (laser recordings) for the different types of body-shaking (BS1-4). Within each type of body-shaking (BS1-4), the values are detailed for each of their parts (P1-4; see Fig. S2–S3 for more details on the parts). Significant differences are indicated by different letters (*p* < 0.05). Dot indicates outliers.

**Figure 6:**
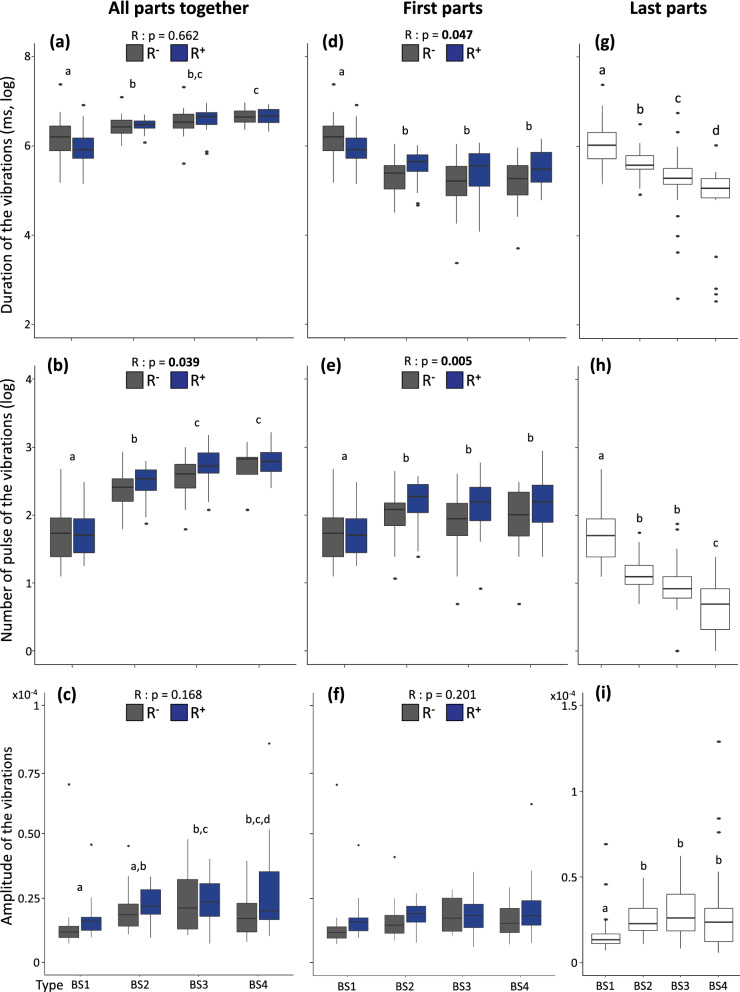
Duration, number of pulses, and amplitude of the vibration events measured by laser recordings. The different types of body-shaking (BS1-4) are represented, in absence of reproductives (R^−^, grey boxes) or in presence of reproductives (R^+^, blue boxes). The *p* values of the reproductive effects are indicated in bold when significant. Data (R^−^ and R^+^) were pooled together (white boxes) when no reproductives effects were found. Note that for representation comparisons, some graphs have not been pooled. Different representations are shown: all parts pooled together i.e. the entire vibration events (**a**–**c**), the first parts only (**d**–**f**), and the last parts only (**g**–**i**) for each type of body-shaking (BS1-4). Within each graph, different letters represent significant differences between each type of body-shaking (*p* < 0.05). Dots indicate outliers.

The frequencies of the produced vibrations were affected by an interaction between the types of the body-shaking and their parts (χ^2^ = 418.47, *p* < 0.0001; Fig. 5). Overall, frequencies present low and high values with two distinctive blocks composed of (1) the last part of each body-shaking types including the first part of the BS1 (which is a first and last part at once) and (2) the other parts (all *p* < 0.0001). The effect of the presence of reproductives and flashlight on frequencies presented complex significant interactions. To clarify those interactions, we investigated separately the duration and the number of pulses. Indeed, a frequency corresponds to the number of pulses divided by the duration of the vibration events. From those investigations, the analyses of the structures of the vibration events (all parts together) revealed a significant influence of the reproductives on the number of pulses (Fig. 6b; χ^2^ = 4.28, *p* = 0.039) but no effect on the total duration (Fig. 6a) or the amplitude (Fig. 6c; see details in Table 1). The reproductives also influenced the first part only of the vibration events (all types together) with an effect on the number of pulses (Fig. 6e; χ^2^ = 7.85, *p* = 0.005) and on the total duration (Fig. 6d; χ^2^ = 3.94, *p* = 0.047), but not on the amplitude (Fig. 6f).

Each type of emitted vibrations presented specificities for the total duration, the number of pulses and the amplitude (Fig. 6, Table 1). Note that the amplitude of a vibration event depends on the distance between the emitter and its receiver. Therefore, amplitude values are difficult to measure with live individuals in a free arena. Nevertheless, because individuals performing vibrations are randomly distributed in the arena, we investigated amplitude values, but data should be treated cautiously. Nevertheless, our analyses showed that over the different parts of each types of body-shaking, only the first and last parts present variabilities, along with the entire vibration event (with all the parts together). Globally, the entire vibration events (all parts together; Fig. 6a–c) show an increase in duration, number of pulses and amplitude when the number of parts increase (from BS1 to BS4). For the first parts only (Fig. 6d–f), the total duration shows a tendency to decrease while the number of pulses increase from BS1 to BS4 (no effect on the amplitude). Finally, the last parts of each types (Fig. 6g–i) decrease in duration and number of pulses but increase in the amplitude from BS1 to BS4. The flashlight had no effect on any structure of the vibration events (all *p* > 0.160) except for one triple interactions (Table 1) only influenced by the second part of BS4, where in absence of reproductives the duration of this second part decreases in presence of a flashlight (χ^2^ = 9.0649, *p* < 0.003).

All the results, represented in the Fig. 6, are sum-up in a schematic representation in the supplementary Fig. 3, except for the amplitude which must be treated cautiously. Indeed, the amplitude is not a cumulative data, and no effect of the presence of the reproductives was detected. The schematic representation clearly shows the different proportions of each parts (Fig. S3). It also reveals that the second and third parts (P2-3) of the different types of body-shaking do not vary in their total duration and number of pulses.

## Discussion

We showed that the vibrations produced by body-shaking behaviour is social context-dependent in *R. flavipes*. The vibrations produced by the body-shaking was not only measurable thanks to the proposed experimental design, but we showed that such vibrations could be transmitted by the substrate between individuals. It calls for more study on the potential function of such behaviour in social communication. Especially since the presence of reproductives affect the duration and the number of pulses of the produced vibrations.

Our results revealed that the occurrence of the body-shaking events increase in presence of reproductives, and very briefly in interactions with the flashlight, a stimulus considered as a stress event in the literature^24,28^. Furthermore, our analyses of the duration and the number of pulses of the vibrations produced by the body-shaking behaviours allowed us to classify them in several types of body-shaking with different specific structures. This diversity in the structure of the emitted vibrations is a pre-request for holding specific information. Interestingly, only the first parts of the emitted vibrations are affected by the presence of reproductives when we look to each part separately, with variation of its duration and number of pulses. Overall, knowing the detailed structure of the produced vibrations could allow to unravel the role of the vibratory behaviours in social interactions.

We describe six different types of body-shaking based on their emitted vibrations, with specificities in duration and number of pulses. A large proportion of body-shaking corresponds to the type BS2 which represent two thirds of the total number of events. Previous study from Whitman and Forschler^23^described four different types of body-shaking (Types 1–4). The authors classified them based on behavioural observations only according to their measured durations, observed frequencies, or the presence of dejections. Direct comparison with this previous work is tedious since the technics used to qualify them are not the same. Indeed, Whitman and Forschler^23^ did direct observations where the current setup allowed to do both behavioural observations and laser analyses, permitting us to link the body-shaking events to precise measurements of the emitted vibrations. Nevertheless, it seems that the duration of the BS1 might correspond to the type I, described by Whitman and Forschler^23^, which is 0.5 s but with high frequency^23^. Type III seems to correspond to BS2, a high frequency part following by a low frequency part with equivalent durations. The type II with longer duration might correspond to the BS3 and BS4 but type II present only low frequency. Whitman and Forscher also described the type IV which is a special long lasting body-shaking of 3 s associated to dejections after a movement^23^. We never observed dejections after a body-shaking event, may be because in our experiments, individuals were sorted on a CO2 pad before to be moved into the tested arena. This first step may sometimes favour defecation during the anaesthesia. Over the different type of body-shaking, BS5 and BS6 were not described by Whitman and Forschler^23^, but even in our study they were present only at a very low occurrence (13 events over 1306). The discrepancies between the two studies could be explained by the experimental design. Indeed, laser vibrometers used here allow more precise measurement of each type of body-shaking with their specific durations and number of pulses. In another study from Delattre et al*.*^9^, they detected the emission of high and low frequencies often performed together, which could be compared to the BS1 and BS2 we observed. The authors quantified vibrations by measuring groups of individuals composed of workers and soldiers. Therefore, emitted vibrations by soldiers, like head-drumming (the individual hit the substrate with the head), were measured along with body-shaking events of soldiers and workers. Moreover, vibration measurements were not directly connected to real-time behavioural observations. Nevertheless, Delattre et al*.*^9^ described two distinctive frequency bursts of 31.0 and 7.4 Hz, which is close to the structure and the frequencies we have found for the BS2 type with a mean frequency for each part of 38.4 ± 6.7 Hz and 13.8 Hz ± 4.0 Hz. However, it does not correspond to the frequencies of BS3 (47.2 ± 22.6 Hz and 25 Hz ± 25.8 Hz) and BS4 (54 ± 27.5 Hz and 26.5 Hz ± 21.3 Hz). Because in Delattre et al*.*^9^, the group were a mixed of workers and soldiers and because emitted vibrations are not directly linked to body-shaking events only, direct comparisons are difficult to draw with our study. Overall, our results show that the produced vibrations are diversified, context dependent, and could be transmitted through the substrate. Moreover, they hold a complexity suitable to allow transmission of information, opening the question of the body-shaking behaviour and its produced vibrations as a communication signal. It should be noted that the vibratory signals measured in our work reflect the substrate response to termite oscillatory motions. The tympan-like paper membrane used in our experiments was intended to minimize signal attenuation. However, in the case of more complex and heterogeneous substrates such as the ones observed in field conditions (i.e., wood, granular media, etc.) the response would be quite different. In order to verify our results in field conditions, further work should include more specific signal processing technics allowing to extract the emitted signal out from the substrate vibratory response^49,50^.

The different types of body-shaking show variations in their occurrence in presence of reproductives and flashlight. It raises again the question of the body-shaking behaviour as a communication signal since the increase in occurrence of some types of body-shaking seems to be dependant of the context (reproductive presence, alarm signal, or both). This diversity in proportions must be added to the complexity of the structure of the signal itself, strengthening even more the diversity holding by this behaviour, increasing the possibility of a role as a signal to transmit information between individuals. Indeed, information could be encoded either through the number of body-shaking events and/or through the structure of the emitted vibrations (number of pulses or durations). In chemical communications, compounds concentration, molecular diversity of the components or even variation of chemicals in a blend are used to transmit and modulate information according to the context^12,51,52^. We could consider that modulation of vibrations could also act the same way as chemicals. Vibrations could also act by themself or in synergy with them^21^. Recently, Sun et al^34^ showed that chemicals and vibrations could be part of a behavioural sequence involved in a reproductive regulation. Therefore, the role played by chemical compounds described in queen or alarm signalling^53,54^ should be studied in the light of the potential implications of vibratory communications^9,55^ as a whole entity, calling for more detailed experiments to unravel the specific role of each cue.

The presence of reproductives affected the structures of the emitted vibrations during a body-shaking event. Precisely, it increased the number of pulses (all parts together and first parts only) and decreased the duration of the first parts, which make sense since duration and number of pulses are cross linked data. It also points out the role that could be attributed to the first parts of the vibrations. In termites, body-shaking have been first described to be involved in alarm behaviours^9,24^ or in cannibalism^41^. But several studies also suggested a function in reproductive signalling^34–36,56^ with a direct modulation by the presence of the reproductives. In other eusocial insects, the presence of other colony members could involve modulation of the vibratory signals in a recruitment context^16,57,58^. Compared to volatile chemicals or of course visual cues, vibration cues possess advantages in subterranean life or in closed environments. Indeed, chemical signals are transmitted less rapidly than vibrations^59^ and they allow to transmit information through walls at long-distances^60^. In fact, vibratory signals are largely widespread but surprisingly neglected by researchers, particularly in insect communication compared to the study of chemical communication^61,62^. Fast transmission of information into the entire colony through walls could be a critical process especially if it encodes for critical social information such as the presence of reproductives or their precise localization in the nest.

Our results reveal that the presence of reproductives increased the occurrence of body-shaking events. This is in accordance with the current literature showing an increase of body-shaking events in presence of reproductives, eggs or even queen pheromones^34–36^ . For now, no relation can be made between this behaviour and the social conflict observed in other termites liked *Cryptotermes secundus*^63^ and *Zootermopsis nevadensis*^64^, where the absence of reproductives induces head-butting between individuals which are candidates for the access to the reproductives status. The increase of body-shaking in presence of reproductives is consistent over time while it is only transitory in presence of a flashlight. Indeed, the number of body-shaking events is higher at short time after the flashlight (here at 15 s only) and only in presence of reproductives. Behaviours induced by a flashlight event is considered as a stress response and seems to be species dependant. It has been studied in different termite species with experimental groups composed of workers and soldiers. The flashlight increases vibratory behaviour in *Incisitermes marginipennis*, *Coptotermes gestroi*, *C. formosanus* and *Zootermopsis angusticollis* but not in *C. niger*^20,24^ and more contrastingly for our studied model *Reticulitermes flavipes*^9,24^ (Delattre et al*.* (2019) measured the speed of motion to characterized the emitted vibrations). Note that in our experimental design, we did not use air puff or vibratory stimuli because they also produce vibrations, which would have distorted our vibrations recordings. If we do not consider discrepancies between the experimental procedures, the main difference is the absence of soldiers in our study. We can hypothesize that the presence of soldier could modify the behavioural responses of the workers like it has been demonstrated in the termite *Hodotermopsis sjostedti*. In this species the defensive behaviours of workers is low in presence of soldiers and high in their absence^65^. The contrary is also true in *Reticulitermes* species, where the absence of workers prevent soldiers to express defensive behaviours including vibrations^22^. Altogether, it shows that vibratory behaviour seems to be influenced by an interaction between the nature of the stimulus and caste composition. It also leads to new questions about other vibratory behaviours (alone or in combination) such as head-drumming^9^ (none were observed in our experimental design) to measure in more details the produced vibrations and their potential implications in social interactions.

Vibroacoustic communication is widespread in social insects and particularly in termites where it is overrepresented. Vibrations produced by body-shaking, head-drumming or resulting from chewing have been previously studied^9,17,56,60,64^. Here, we show that body-shaking produce vibrations transmitted to the substrate that could be pursued by other colony members. Those emitted vibrations could be involved in different contexts due to their diversity, raising interesting questions on their functions. If it is alarming, how does it work? If it tells the presence of reproductives, why workers would transmit this to other individuals? This seemingly simple behaviour produces vibrations which can deliver messages at fast speed throughout physical obstacles. We discovered that those vibrations were diverse and could be modulated with: (1) the occurrence of body-shaking events; (2) the variation in duration or number of pulses of each emitted vibration; (3) and the diversity of each type of body-shaking with their own specificities. All those characteristics make body-shaking behaviour suitable to encode complex information, as good as chemical signals which are considered as the main cues in insect communications. Potential implication of the vibratory behaviours in royal recognition is a growing interesting topic, but vibratory behaviours might also be involved in other social interactions. Because vibrations are used by termites for food choice, workers attraction, and reproductive reguation^17,56^ it would be interesting to investigate the possible implication of the body-shaking behaviour in caste differentiation or social signalling.

## Supplementary Information

Supplementary Figures.

## Data Availability

Data are available from the Zenodo Digital Repository: 10.5281/zenodo.4733545.
