# Supplementary material for: Vibratory behaviour produces different vibration patterns in presence of reproductives in a subterranean termite species
Source: Sci Rep. 2021 May 10;11:9902. doi: 10.1038/s41598-021-88292-7 (PMC8110524; doi:10.1038/s41598-021-88292-7)
Supplement: Supplementary file 1 — Supplementary Figures. [file 41598_2021_88292_MOESM1_ESM.pptx]

## Slide 1
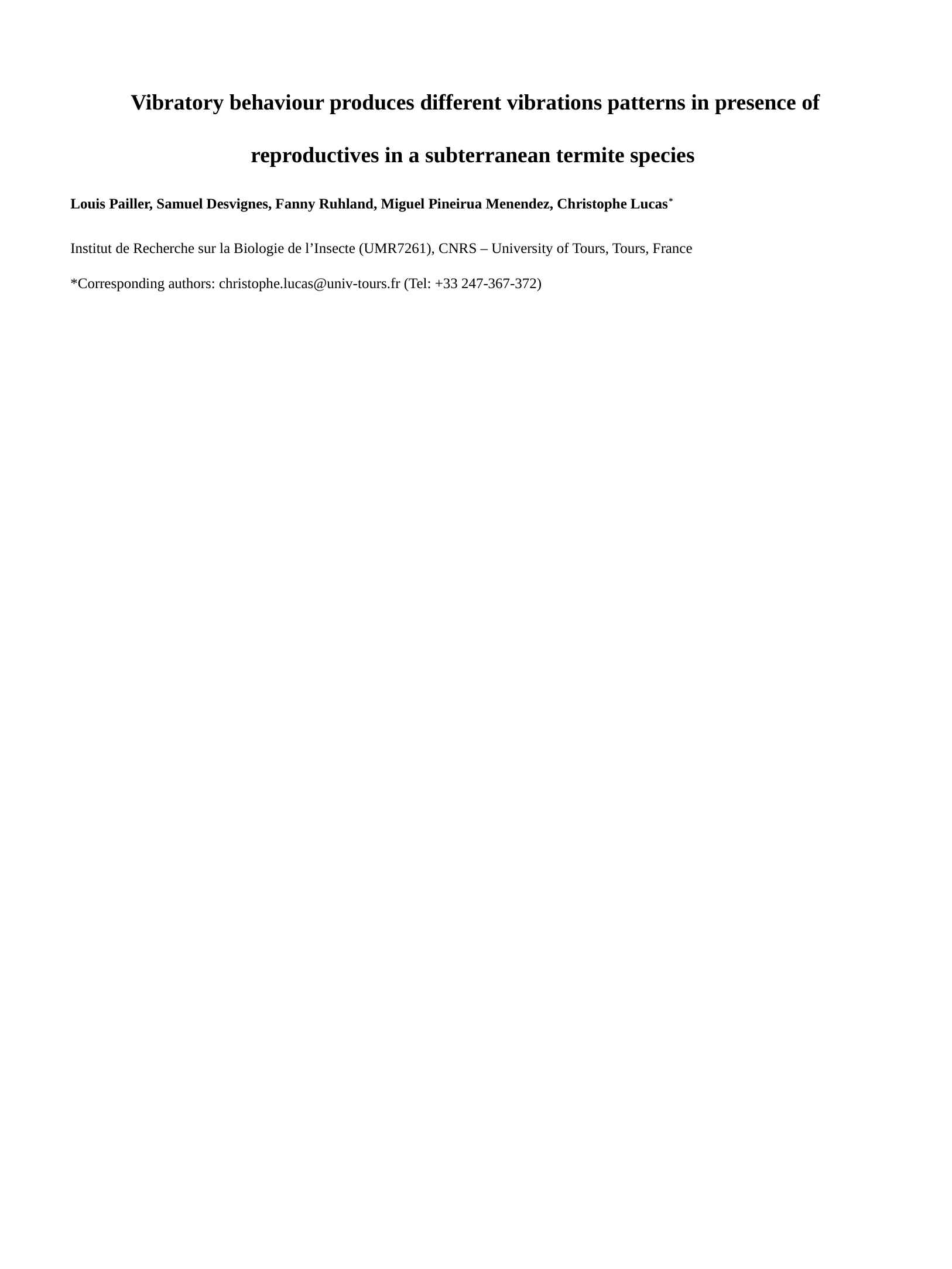

Vibratory behaviour produces different vibrations patterns in presence of reproductives in a subterranean termite species
Louis Pailler, Samuel Desvignes, Fanny Ruhland, Miguel Pineirua Menendez, Christophe Lucas*
Institut de Recherche sur la Biologie de l’Insecte (UMR7261), CNRS – University of Tours, Tours, France
*Corresponding authors: christophe.lucas@univ-tours.fr (Tel: +33 247-367-372)

## Slide 2
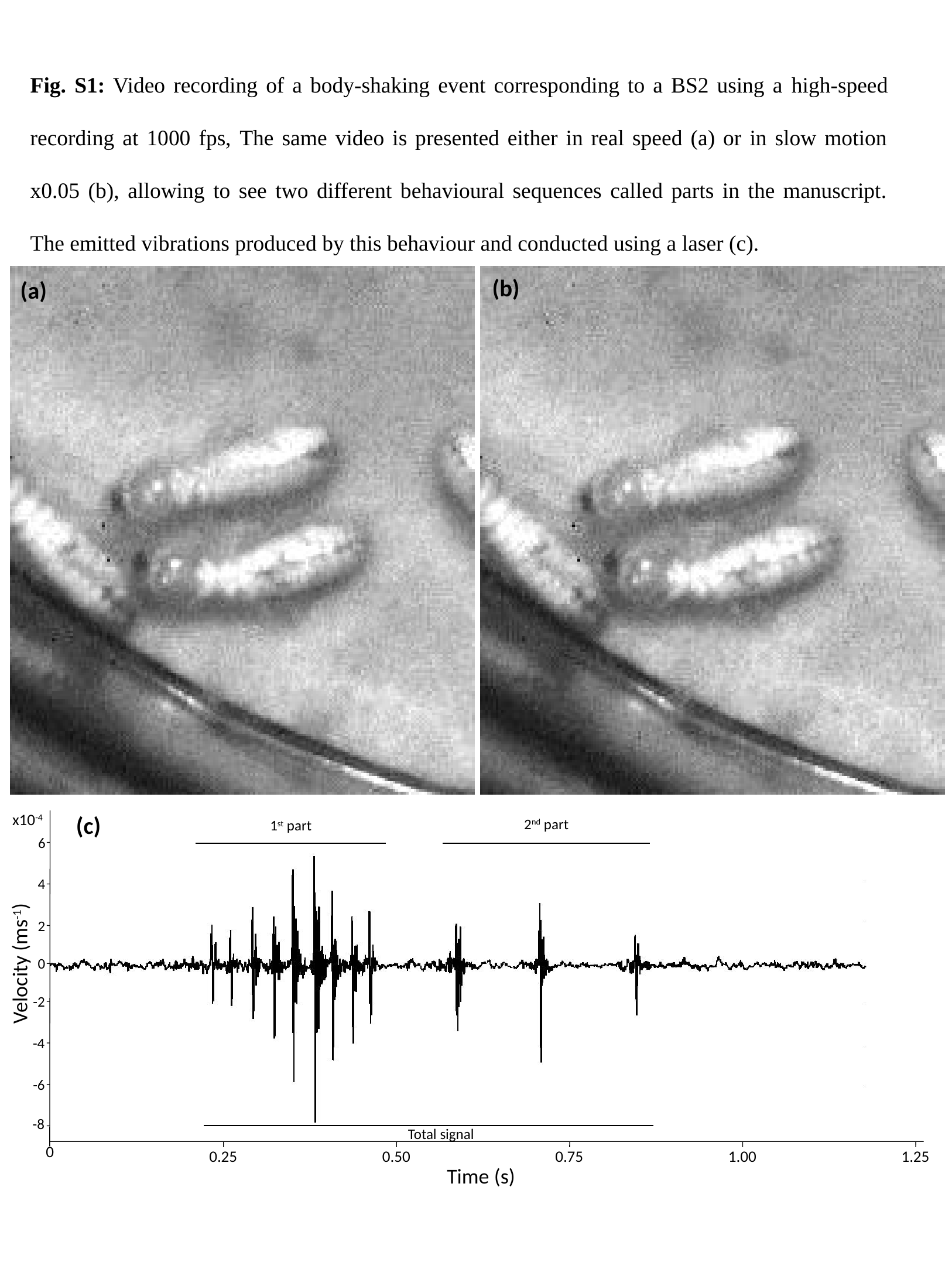

Fig. S1: Video recording of a body-shaking event corresponding to a BS2 using a high-speed recording at 1000 fps, The same video is presented either in real speed (a) or in slow motion x0.05 (b), allowing to see two different behavioural sequences called parts in the manuscript. The emitted vibrations produced by this behaviour and conducted using a laser (c).
(b)
(a)
(c)
x10-4
6
4
2
0
Velocity (ms-1)
-2
-4
-6
0
0.25
0.50
0.75
1.00
1.25
Time (s)
-8
2nd part
1st part
Total signal

## Slide 3
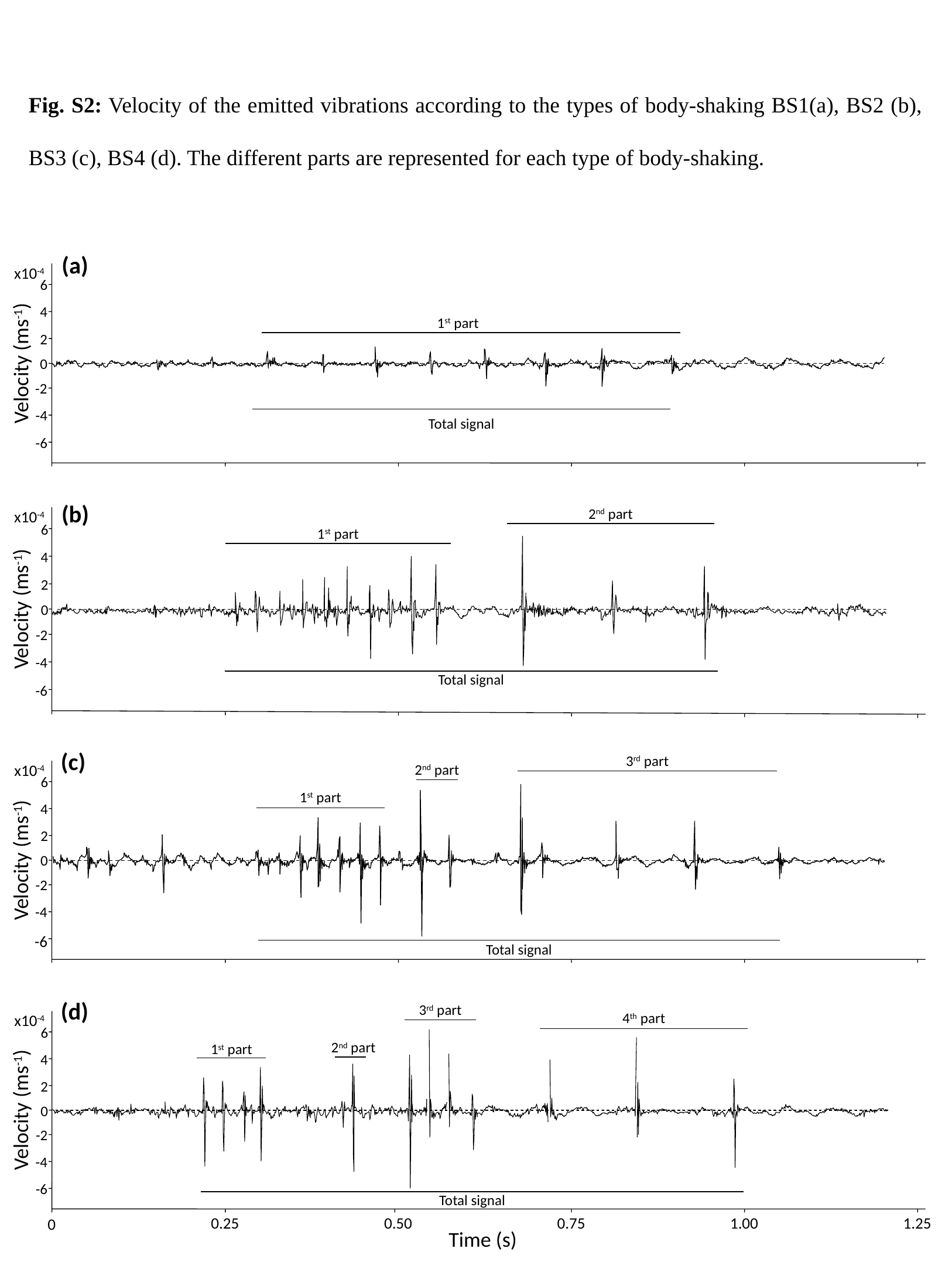

Fig. S2: Velocity of the emitted vibrations according to the types of body-shaking BS1(a), BS2 (b), BS3 (c), BS4 (d). The different parts are represented for each type of body-shaking.
(a)
x10-4
6
4
2
Velocity (ms-1)
0
-2
-4
Total signal
-6
1st part
(b)
2nd part
x10-4
1st part
6
4
2
0
-2
-4
Total signal
-6
Velocity (ms-1)
(c)
x10-4
6
4
2
0
-2
-4
-6
Total signal
Velocity (ms-1)
3rd part
2nd part
1st part
(d)
3rd part
4th part
x10-4
6
2nd part
1st part
4
2
Velocity (ms-1)
0
-2
-4
-6
Total signal
0.25
0.50
0.75
1.00
1.25
0
Time (s)

## Slide 4
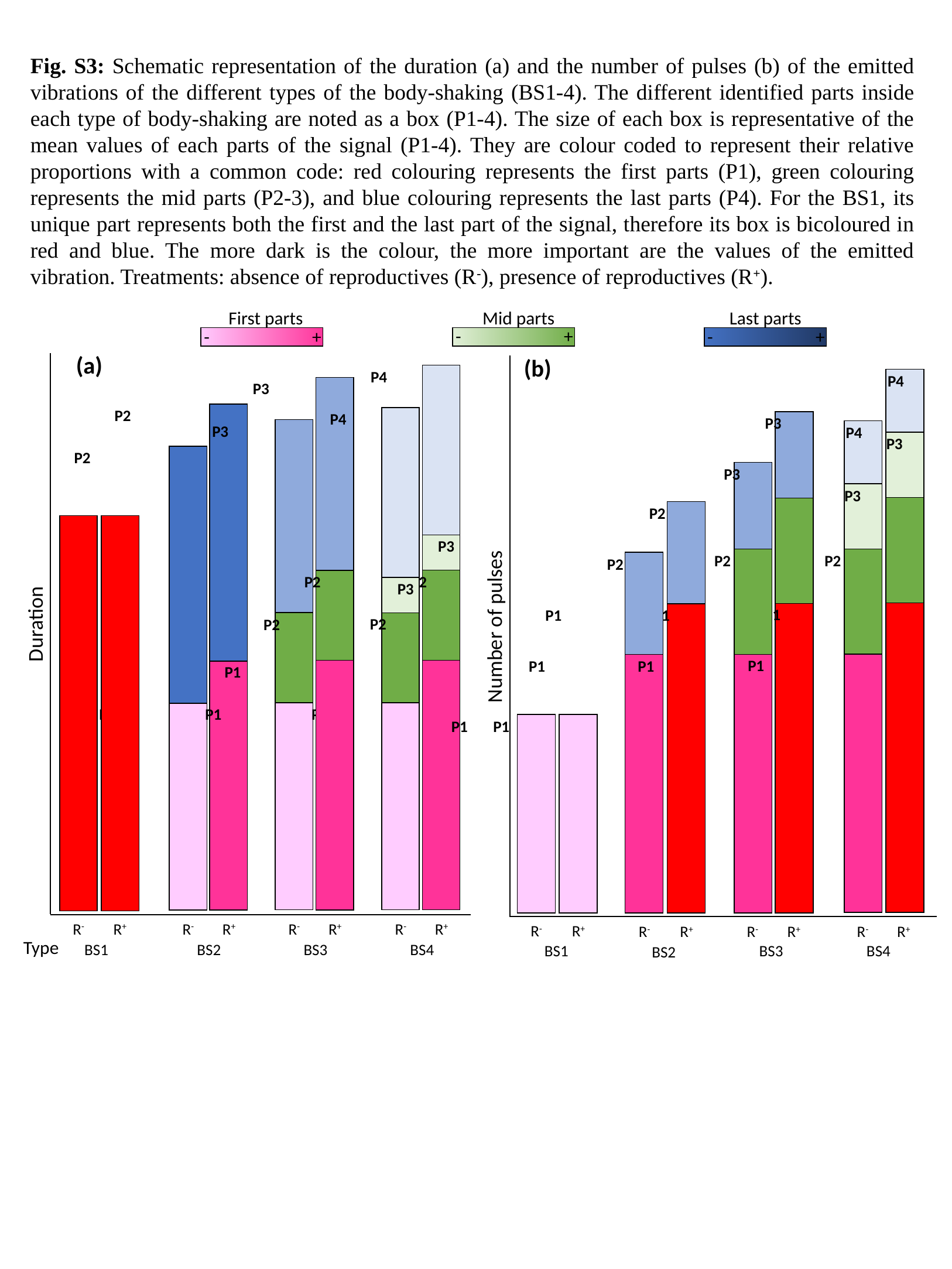

Fig. S3: Schematic representation of the duration (a) and the number of pulses (b) of the emitted vibrations of the different types of the body-shaking (BS1-4). The different identified parts inside each type of body-shaking are noted as a box (P1-4). The size of each box is representative of the mean values of each parts of the signal (P1-4). They are colour coded to represent their relative proportions with a common code: red colouring represents the first parts (P1), green colouring represents the mid parts (P2-3), and blue colouring represents the last parts (P4). For the BS1, its unique part represents both the first and the last part of the signal, therefore its box is bicoloured in red and blue. The more dark is the colour, the more important are the values of the emitted vibration. Treatments: absence of reproductives (R-), presence of reproductives (R+).
First parts
Mid parts
Last parts
- +
- +
- +
(a)
P4
P3
P4
P3
P2
P3
P2
P3
P2
P2
Duration
P2
P2
P1
P1
P1
P1
P1
P1
P1
P1
R-
R+
R-
R+
R-
R+
R-
R+
Type
BS1
BS2
BS3
BS4
(b)
P4
P4
P3
P3
P3
P3
P2
P2
P2
P2
P2
P2
Number of pulses
P1
P1
P1
P1
P1
P1
P1
P1
R-
R+
R-
R+
R-
R+
R-
R+
BS3
BS4
BS1
BS2
